# Supplementary material for: Biomarker-Based Assessment Model for Detecting Sepsis: A Retrospective Cohort Study
Source: J Pers Med. 2023 Jul 27;13(8):1195. doi: 10.3390/jpm13081195 (PMC10455581; doi:10.3390/jpm13081195)
Supplement: Supplementary file 1 [file jpm-13-01195-s001.zip › jpm-2462528-supplementary.pdf]

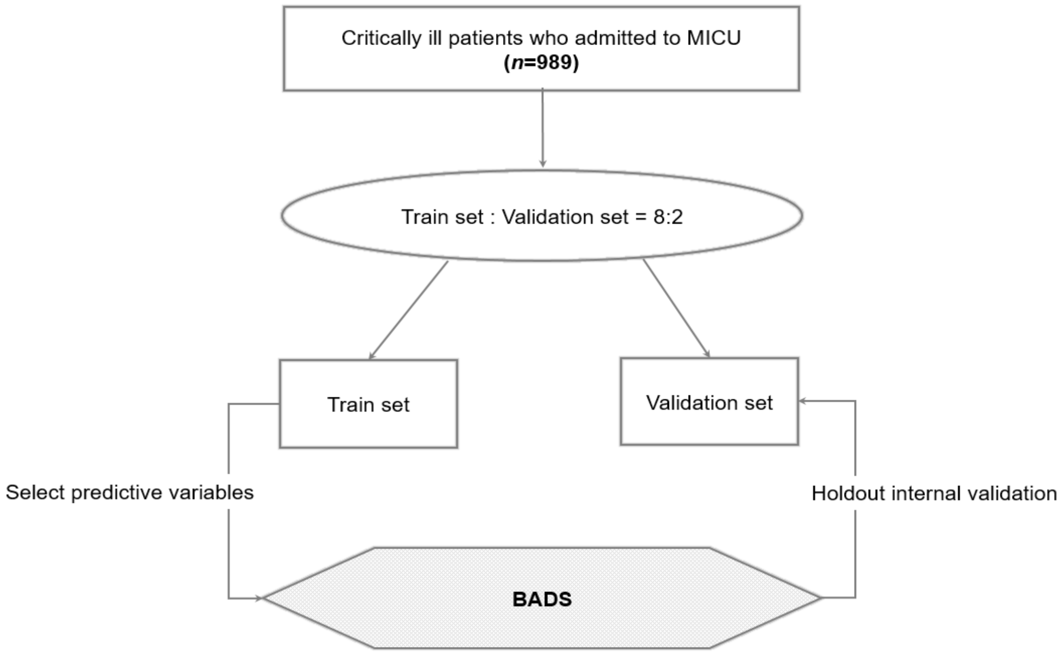

Supplementary Figure S1. Study flow for the development and validation of Biomarker-based Assessment model for Detecting Sepsis (BADS) score.

Supplementary Table S1. Baseline characteristics in the training cohort

	**Non-sepsis** n=180 (22.8 %)	**Sepsis** n=611 (77.2 %)	***p* Value**
Age (year)	64 (55, 75)	69 (57, 77)	0.056
Sex (male), n (%)	101 (56.1)	395 (64.6)	0.044
BMI (kg/m^2)	22.2 (19.4, 24.3)	22.1 (19.5, 24.8)	0.634
qSOFA score	1 (1, 2)	2 (1, 3)	<0.001
SOFA score	6 (3, 9)	10 (7, 13)	<0.001
APACHE II score	19 (14, 27)	26 (19, 32)	<0.001
CCI	3 (2, 5)	3 (2, 5)	0.515
Mean arterial pressure (mmHg)	80 (66-108)	67 (55-80)	<0.001
Systolic blood pressure (mmHg)	96 (76-113)	86 (72-102)	<0.001
Heart rate (beats/min)	97 (80-112)	107 (88-126)	<0.001
Shock index	1.03 (0.73-1.36)	1.27 (0.91-1.63)	<0.001
Lactate (mmol/L)	1.2 (0.9-2.2)	2.4 (1.4-5.0)	<0.001
Procalcitonin (ng/mL)	0.54 (0.20-1.85)	1.8 (0.4-13.1)	<0.001
ARDS, n (%)	10 (5.6)	71 (11.6)	0.017
AKI, n (%)	46 (25.6)	202 (33.1)	0.067
Positive blood culture, n (%)	16 (8.9)	216 (35.4)	<0.001
28 days mortality, n (%)	36 (20.0)	214 (35.0)	<0.001

Values are expressed as n (%) or median (interquartile range) unless otherwise indicated. BMI, body mass index; qSOFA, quick Sequential Organ Failure Assessment; SOFA, Sequential Organ Failure Assessment; APACHE II, Acute Physiology and Chronic Health Evaluation Score II; CCI, Charlson Comorbidity Index; ARDS, acute respiratory distress syndrome; AKI, acute kidney injury

Supplementary Table S2. Baseline characteristics in the validation cohort

	**Non-sepsis** n=44 (22.2 %)	**Sepsis** n=154 (77.8 %)	***p* Value**
Age (year)	64 (56, 75)	67 (56, 76)	0.578
Sex (male), n (%)	31 (70.5)	92 (59.7)	0.221
BMI (kg/m^2)	22.2 (19.4, 24.3)	21.9 (19.4, 24.7)	0.177
qSOFA score	1 (1, 2)	2 (1, 2)	0.003
SOFA score	4 (3, 8)	10 (8, 12)	<0.001
APACHE II score	19 (15, 26)	24 (18, 33)	<0.001
CCI	3 (2, 4)	3 (1, 4)	0.434
Mean arterial pressure (mmHg)	76 (63-84)	65 (55-75)	0.049
Systolic blood pressure (mmHg)	99 (86-119)	84 (72-101)	0.001
Heart rate (beats/min)	86 (74-105)	104 (89-124)	0.001
Shock index	0.85 (0.64-1.20)	1.21 (0.87-1.67)	<0.001
Lactate (mmol/L)	1.30 (1.00-2.30)	2.50 (1.60-5.33)	0.002
Procalcitonin (ng/mL)	0.50 (0.20-1.40)	1.95 (0.43-18.65)	<0.001
ARDS, n (%)	3 (6.8)	15 (9.7)	0.768
AKI, n (%)	8 (18.2)	52 (33.8)	0.062
Positive blood culture, n (%)	2 (4.5)	57 (37.0)	<0.001
28 days mortality, n (%)	6 (13.6)	52 (33.8)	0.009

Values are expressed as n (%) or median (interquartile range) unless otherwise indicated. BMI, body mass index; qSOFA, quick Sequential Organ Failure Assessment; SOFA, Sequential Organ Failure Assessment; APACHE II, Acute Physiology and Chronic Health Evaluation Score II; CCI, Charlson Comorbidity Index; ARDS, acute respiratory distress syndrome; AKI, acute kidney injury

Supplementary **Table S3.** Logistic regression analysis for risk factors of sepsis

Train set (N=791)	Univariable logistic			VIF	Missing	Multivariable logistic	
	OR (95% CI)	p-value	AUC (95% CI) (%)			OR (95% CI)	p-value
Age	1.012 (1.000, 1.024)	0.0478	54.85(49.97%, 59.74%)	1.12	0		
Sex			52.81(48.66%, 56.96%)	1.07	0		
Male	Ref						
Female	0.790 (0.560, 1.113)	0.1778					
BMI	1.010 (0.971, 1.051)	0.6064	51.18(46.37%, 56.00%)	1.14	0		
Culture			63.79(60.96%, 66.62%)	1.21	0		
Negative	Ref					Ref	
Positive	5.988 (3.440, 10.425)	<.0001				4.662(2.197, 9.890)	0.0001
SBP_D0	0.982 (0.975, 0.988)	<.0001	62.31(57.41%, 67.21%)	3.00	0	0.991 (0.982, 1.000)	0.0557
HR_D0	1.013 (1.007, 1.019)	<.0001	61.12(56.60%, 65.64%)	2.90	0		
RR_D0	1.022 (0.998, 1.046)	0.0691	54.78(49.72%, 59.85%)	1.20	0		
BT_D0	1.018 (0.882, 1.176)	0.8058	51.05(46.24%, 55.85%)	1.31	0		
MAP_D0	0.982 (0.976, 0.988)	<.0001	67.11(62.66%, 71.56%)	1.29	0	0.987 (0.979, 0.995)	0.0025
OU_D0	1.000 (1.000, 1.000)	0.0585	53.60(48.63%, 58.57%)	1.47	0		
GCS_D0	0.948 (0.913, 0.984)	0.0055	56.36(52.05%, 60.66%)	1.23	0		
Glu_D0	1.003 (1.001, 1.005)	0.0089	57.23(52.64%, 61.83%)	1.08	0	1.003 (1.000, 1.006)	0.0986
CRP_D0	1.006 (1.004, 1.008)	<.0001	66.00(61.39%, 70.60%)	1.31	5		
PCT_D0	1.113 (1.060, 1.169)	<.0001	68.62(64.30%, 72.93%)	1.31	71	1.078 (1.032, 1.125)	0.0007
WBC_D0	1.049 (1.027, 1.071)	<.0001	63.62(59.41%, 67.82%)	1.07	0		
Hct_D0	0.991 (0.967, 1.017)	0.5058	51.88(47.07%, 56.68%)	1.49	0		
RDW_D0	1.037 (0.969, 1.109)	0.2982	52.26(47.41%, 57.11%)	1.37	0		
Plt_D0	0.998 (0.997, 0.999)	0.0053	58.60(54.01%, 63.19%)	1.28	0		
DNI_D0	1.096 (1.060, 1.133)	<.0001	69.37(65.17%, 73.56%)	1.45	0		
BUN_D0	0.999 (0.994, 1.005)	0.8068	54.05(48.82%, 59.28%)	2.10	0		
Cr_D0	0.948 (0.883, 1.018)	0.1423	52.52(47.44%, 57.61%)	2.15	0		
Alb_D0	0.355 (0.257, 0.490)	<.0001	65.37(60.69%, 70.05%)	1.40	0	0.438 (0.282, 0.681)	0.0002
TB_D0	1.051 (0.995, 1.110)	0.0774	58.24(53.68%, 62.81%)	1.27	0		
Na_D0	1.034 (1.010, 1.058)	0.0055	56.34(51.67%, 61.01%)	1.22	0	1.039 (1.004, 1.075)	0.0268
K_D0	0.945 (0.801, 1.114)	0.5012	54.53(49.92%, 59.15%)	1.29	0		
Lac_D0	1.232 (1.126, 1.348)	<.0001	71.44(66.63%, 76.25%)	1.43	95	1.097 (1.006, 1.198)	0.0372
PF_D0	0.997 (0.996, 0.998)	<.0001	60.89(56.20%, 65.57%)	1.23	0	0.997 (0.995, 0.999)	0.0002
Shock_index	2.688 (1.864, 3.876)	<.0001	64.26(59.71%, 68.81%)	4.96	0	2.457 (1.844, 3.981)	0.0009

BMI, body mass index; SBP, systolic blood pressure; HR, hazard ratio; RR, respiratory rate; BT, body temperature; MAP, mean arterial pressure; OU, urine output; GCS, Glasgow coma scale; Glu, glucose; CRP, C-reactive protein; PCT, procalcitonin; WBC, white blood cell; Hct, hematocrit; RDW, red cell distribution width; Plt, platelet; DNI, delta neutrophil index; Cr, creatinine; Alb, albumin; TB, total bilirubin; Lac, lactate; PF, the ratio of arterial oxygen partial pressure to fractional inspired oxygen; OR, odds ratio; CI, confidence interval; AUC, area under the curve; VIF, variance inflation factor
